# A New Circulation

**Published:** 1865-09

**Authors:** 


A New Circulation.—Dr. Bence Jones, in a lecture recently delivered (says the *Boston Med. Jour.*) at the Royal Institution, says there are good grounds for believing that there exists within us, in addition to the mechanical or animal circulation of the blood, another and a greater and more strictly chemical circulation, closely resembling, if not identical with, that which obtains in the lower divisions of animals, and in vegetables. A circulation in which substances continually pass from the outside of the body into the blood, and through the blood into the textures, and from the textures either into the ducts, by which they again pass into the blood, or are thrown out of the body; or into the absorbents, by which they are again taken back into the blood, again to pass from it into the textures.

